# Activation of β-Adrenoceptors Promotes Lipid Droplet Accumulation in MCF-7 Breast Cancer Cells via cAMP/PKA/EPAC Pathways

**DOI:** 10.3390/ijms24010767

**Published:** 2023-01-01

**Authors:** Dany Silva, Katarzyna Kacprzak, Clara Quintas, Jorge Gonçalves, Paula Fresco

**Affiliations:** 1 Laboratory of Pharmacology, Department of Drug Sciences, Faculty of Pharmacy, University of Porto, 4050-313 Porto, Portugal; 2 UCIBIO-REQUIMTE, Department of Drug Sciences, Faculty of Pharmacy, University of Porto, 4050-313 Porto, Portugal

**Keywords:** lipid droplets, breast cancer, β-adrenoceptors, tumourigenesis

## Abstract

Physiologically, β-adrenoceptors are major regulators of lipid metabolism, which may be reflected in alterations in lipid droplet dynamics. β-adrenoceptors have also been shown to participate in breast cancer carcinogenesis. Since lipid droplets may be seen as a hallmark of cancer, the present study aimed to investigate the role of β-adrenoceptors in the regulation of lipid droplet dynamics in MCF-7 breast cancer cells. Cells were treated for up to 72 h with adrenaline (an endogenous adrenoceptor agonist), isoprenaline (a non-selective β-adrenoceptor agonist) and salbutamol (a selective β_2_-selective agonist), and their effects on lipid droplets were evaluated using Nile Red staining. Adrenaline or isoprenaline, but not salbutamol, caused a lipid-accumulating phenotype in the MCF-7 cells. These effects were significantly reduced by selective β_1_- and β_3_-antagonists (10 nM atenolol and 100 nM L-748,337, respectively), indicating a dependence on both β_1_- and β_3_-adrenoceptors. These effects were dependent on the cAMP signalling pathway, involving both protein kinase A (PKA) and cAMP-dependent guanine-nucleotide-exchange (EPAC) proteins: treatment with cAMP-elevating agents (forskolin or 8-Br-cAMP) induced lipid droplet accumulation, whereas either 1 µM H-89 or 1 µM ESI-09 (PKA or EPAC inhibitors, respectively) abrogated this effect. Taken together, the present results demonstrate the existence of a β-adrenoceptor-mediated regulation of lipid droplet dynamics in breast cancer cells, likely involving β_1_- and β_3_-adrenoceptors, revealing a new mechanism by which adrenergic stimulation may influence cancer cell metabolism.

## 1. Introduction

The sympathetic nervous system plays a prominent role in the neural regulation of breast cancer progression [1,2,3,4]. These sympathetic effects have been ascribed, mainly, to the activation of β-adrenoceptors [1,2,3,4,5] by the adrenergic transmitters noradrenaline and adrenaline. Both cancer and non-cancer cells present in the tumour microenvironment express β-adrenoceptors, and their activation influences multiple cellular processes involved in the progression of breast cancer [3,6,7,8,9,10]. These β-adrenoceptor-mediated effects have an impact on the response to cancer therapies. In fact, the activation of β-adrenoceptors in the tumour microenvironment has been linked to impairments in anti-tumour responses and to cancer cell resistance to several chemo- and targeted therapies [1,11,12,13,14,15], decreasing the efficacy of the treatments for breast cancer. Taking into consideration these prominent roles of the adrenergic system in regulating breast cancer, insights into how it can affect breast cancer are needed in order to better understand the therapeutic potential of targeting this pathway in the treatment of breast cancer.

In addition to the well-known alteration of glucose metabolism observed in cancer cells (the Warburg effect), recent studies have shown that lipid metabolism is also critical for tumorigenesis and cancer progression [16,17]. In lipid metabolism, lipid droplets (LDs) are critical organelles, presenting remarkable dynamics with periods of expansion and shrinkage in response to nutrient fluctuations and various microenvironmental stress conditions [16,18,19]. When fatty acids (FAs) and cholesterol are abundant in the cytoplasm, these neutral lipids are stored in LDs (LD expansion) [20]. However, under high cell metabolic needs, LDs can be important cell sources of FAs, as they release these nutrients (LD shrinkage) [20]. Alterations in the dynamics of LDs have also been shown to be important by fuelling several cancer-related processes, including cell growth [16], stemness [21] and immunity [22], and by impacting the efficacy of anti-cancer drugs [23].

In a physiological context, the adrenergic system plays a major role in the regulation of LD dynamics (LD expansion and shrinkage) [24,25,26,27]. In adipocytes, typical FA storage cells, the adrenergic regulation of lipolysis is activated under negative energy balance conditions, such as fasting and intensive physical exercise [24,25]. Under these circumstances, the activation of β-adrenoceptors stimulates the recruitment of FAs from adipocyte LDs into the blood circulation in order to correct the negative energy balance. This β-adrenoceptor-mediated mobilisation of FAs from adipocyte LDs involves the activation of protein kinases, particularly protein kinase A (PKA), which phosphorylates the LD-associated protein perilipin-1 (PLIN-1), rendering LDs susceptible to lipolysis [28]. Phosphorylated PLIN-1 allows adipose triglyceride lipase (ATGL) to access triacylglycerols (TAGs), with the consequent formation of diacylglycerol (DAG) and free FAs [25,29,30]. The cytoplasmic hormone-sensitive lipase (HSL) is also phosphorylated by PKA, leading to its translocation to the surface of the LDs, where it catalyses the subsequent release of a FA from the DAG generated by the ATGL activity [31,32,33].

Some authors have suggested that the β-adrenoceptor regulation of LD dynamics can be different in non-adipose tissues (more oriented towards promoting FA retention, probably to address higher energetic needs) [26,27,34,35]. In skeletal muscle, the adrenoceptor-mediated induction of TAG storage associated with myofiber remodelling has been reported [35]. A β-adrenoceptor-mediated increase in the number of LDs has also been shown to occur in macrophages [26], astrocytes [27] and hepatocytes [34].

In breast tissues, marked alterations in lipid metabolism and in LD dynamics occur during lactation [36,37], a process that is also under adrenoceptor modulation [38]. In breast cancer, the dysregulation of LD dynamics is often observed, and LD abundance has been positively correlated with cancer progression [16,18,21,39,40]. For instance, LD expansion has been shown to protect breast cancer cells against nutrient stress conditions [18,39,40] and to increase cell survival during hypoxia [16]. These pathways allow cancer cells to shift their survival metabolism according to nutrient fluctuations. LDs have also been reported to provide protection against oxidative stress and cell death [18]. An increase in the number of LDs has also been found to be associated with breast cancer cell stemness [21] and resistance to cancer therapies (e.g., to tamoxifen [41] and to radiation [42]).

Despite the established physiological role of β-adrenoceptors in the regulation of LD dynamics and the fact that LDs have been associated with critical functions in breast cancer cells [16,18,21,39,40], the influence of β-adrenoceptor activation on the dynamics of LDs in cancer cells has not been previously explored. If confirmed, it would provide an additional mechanism to explain the putative protective effect of β-adrenoceptor antagonists in reducing breast cancer progression [43,44]. Therefore, the present study aimed to investigate whether β-adrenoceptor activation alters the dynamics of LDs in MCF-7 breast cancer cells and to explore its underlying transduction mechanisms.

## 2. Results

### 2.1. β-Adrenoceptor Activation Triggers LD Expansion in MCF-7 Breast Cancer Cells

To investigate whether and how β-adrenoceptor activation affects LD dynamics in MCF-7 breast cancer cells, cells were incubated for different incubation periods (24 h and 72 h) with adrenaline, an endogenous adrenoceptor agonist; isoprenaline, a non-selective β-adrenoceptor agonist; and with salbutamol, a selective β_2_-adrenoceptor agonist.

**Figure 1:**
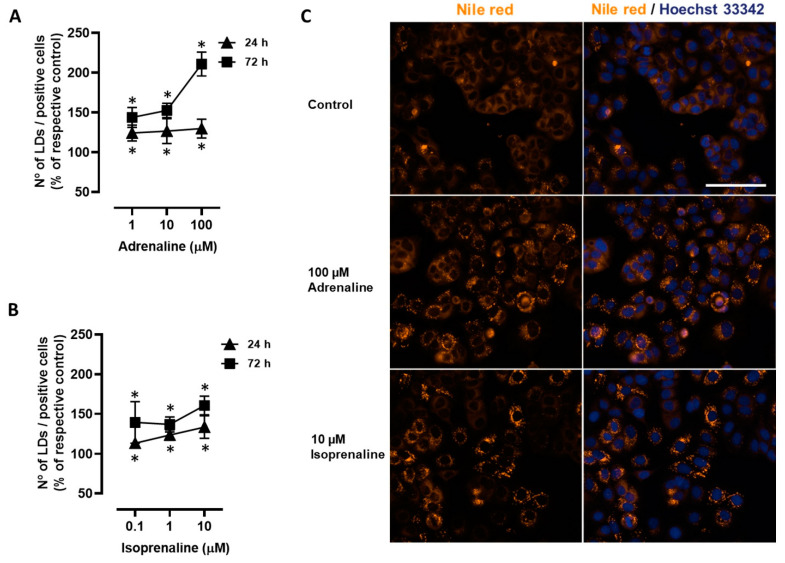
Influence of adrenoceptor activation on the number of lipid droplets (LDs)/positive cells in human MCF-7 breast cancer cells. Cells were treated with either (**A**) the non-selective adrenoceptor agonist adrenaline (1–100 µM) or (**B**) the non-selective β-adrenoceptor agonist isoprenaline (0.1–10 µM) for 24 h and 72 h. Results are expressed as percentage of control (solvent) and are presented as mean ± SD from 4−5 independent experiments. * *p* < 0.05, significantly different from solvent. (**C**) Representative microscopic images of LDs (orange fluorescence) in MCF-7 breast cancer cells without any treatment and after treatment, for 72 h, with either 100 µM adrenaline or 10 µM isoprenaline. Nuclei (blue fluorescence) were labelled with Hoechst 33342. Scale bar: 100 µm.

After the 24 h incubation period with adrenaline (1–100 µM), a significant increase in the number of LDs/positive cells was observed (Figure 1A). The increase in the number of LDs/positive cells, caused by adrenaline, seems to be time-dependent, as the number of LDs/positive cells after the 72 h incubation period was significantly higher than that observed after the 24 h incubation period (Figure 1A: 100 µM, *p* < 0.0001). Isoprenaline (0.1–10 µM) also significantly increased the number of LDs/positive cells, at both incubation periods (Figure 1B). Salbutamol (0.1–10 µM), a selective β_2_-adrenoceptor agonist, did not alter the number of LDs/positive cells (Figure S1A). The number of LDs/positive cells in the presence of 10 µM salbutamol after the 24 h incubation period was 99.6 ± 6.4%, whereas after the 72 h incubation period, it was 101.6 ± 6.8%. Representative images of Nile Red- and Hoechst 33342-stained MCF-7 cells treated with either adrenaline (100 µM) or isoprenaline (10 µM) are shown in Figure 1C. Representative images of salbutamol-treated cells are shown in Figure S1B. Taken together, the present results indicate that β-adrenoceptor activation increases the number of LDs in MCF-7 cells.

### 2.2. Adrenergic Stimulation Induces Alterations in LD Size and LD Loading Capacity

**Figure 2:**
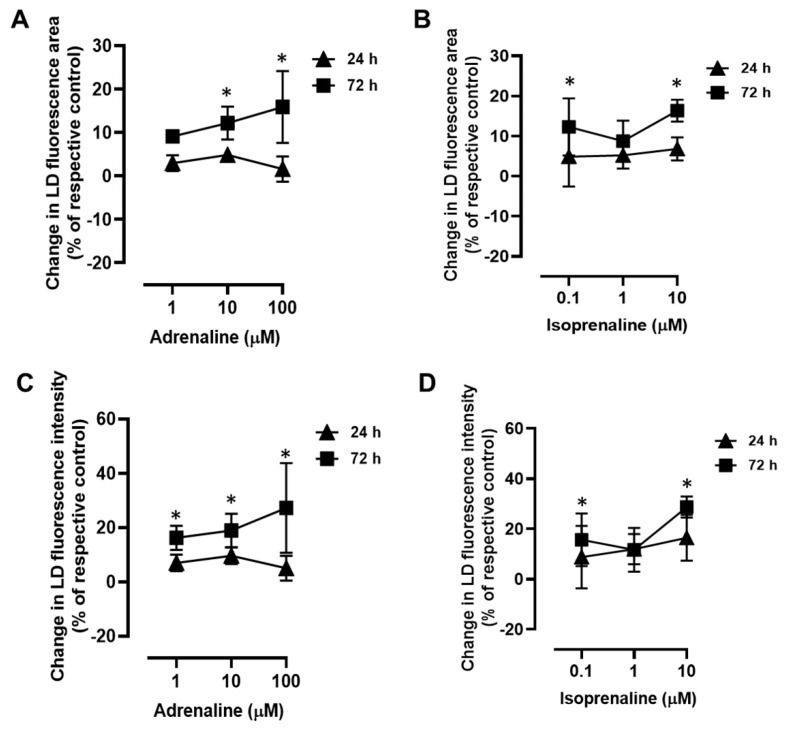
Influence of β-adrenoceptor activation in lipid droplet (LD) area (**A**,**B**) and Nile Red fluorescence intensity (**C**,**D**) in MCF-7 breast cancer cells. Cells were treated with either (**A**,**C**) the non-selective adrenoceptor agonist adrenaline (1–100 µM) or (**B**,**D**) the non-selective β-adrenoceptor agonist isoprenaline (0.1−10 µM) for 24 h and 72 h. Results shown are a percentage of change in LD area or in LD fluorescence intensity compared to control (solvent) and are presented as mean ± SD from 4−5 independent experiments. * *p* < 0.05, significantly different from solvent, one-way ANOVA, post hoc multi-comparisons Dunnett’s test.

To further dissect the influence of adrenergic activation on the dynamics of LDs, the area and fluorescence intensity of the Nile Red-stained LDs (indicators of LD area and LD neutral lipid content, respectively) were also evaluated. Both adrenaline (1–100 µM) and isoprenaline (0.1–10 µM) induced significant increases in the LD area (Figure 2A,B, respectively) and fluorescence intensity after 72 h of incubation (Figure 2C,D, respectively). The β_2_-adrenoceptor agonist, salbutamol, failed to change both the area and fluorescence intensity of the LDs at the two incubation periods: the percentage of change in the LD area in the presence of 10 µM salbutamol after the 24 h incubation period was 3.4 ± 3.0%, whereas after the 72 h incubation period, it was 2.9 ± 5.7%; the LD intensity after the 24 h incubation period was 5.4 ± 4.6%, whereas after the 72 h incubation period, it was 7.7 ± 13.1% (Figure S2A,B, respectively).

### 2.3. Adrenergic-Induced LD Expansion Is Mediated by β_1_- and β_3_-Adrenoceptor Activation

**Figure 3:**
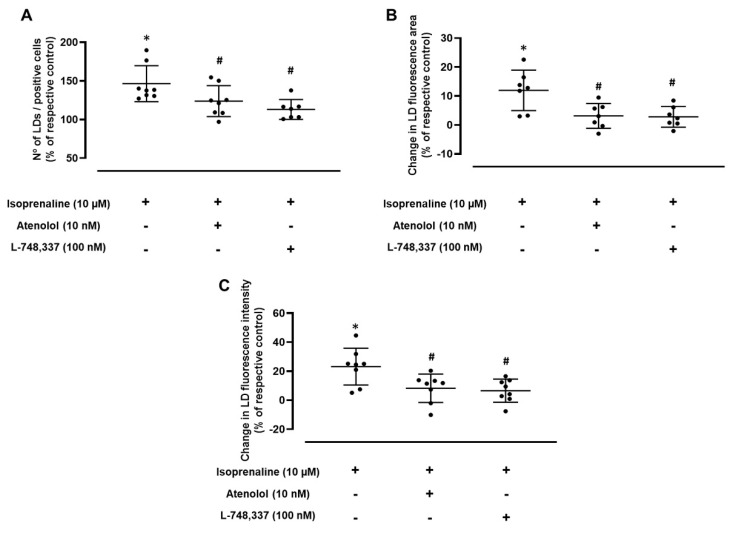
Influence of the selective β_1_-adrenergic receptor antagonist atenolol (10 nM) and the selective β_3_-adrenergic receptor antagonist L-748,337 (100 nM) on the effect caused by isoprenaline (10 µM) on (**A**) the number of lipid droplets (LDs)/positive cells, (**B**) their area and (**C**) their Nile Red fluorescence intensity after 72 h of incubation. Results are expressed as percentage of control (solvent) and are presented as mean ± SD from 7−8 independent experiments. * *p* < 0.05, significantly different from solvent; ^#^ *p* < 0.05, significantly different from isoprenaline treatment.

In the absence of the effects of the selective β_2_-adrenoceptor agonist, salbutamol, the putative contributions of the other β-adrenoceptor subtypes (β_1_ and β_3_) to the LD dynamics were further investigated. Experiments were carried out using isoprenaline, a β-adrenoceptor agonist, and a 72 h incubation period, as these were the experimental conditions where the highest increase in the number of LDs/positive cells was observed. Isoprenaline (10 µM) was tested in the absence and in the presence of atenolol (10 nM) or L-748,337 (100 nM), concentrations described to provide the selective antagonism of β_1_- and β_3_-adrenoceptors, respectively. As shown in Figure 3A, the increase in the number of LDs/positive cells caused by isoprenaline was significantly inhibited by both atenolol and L-748,337, suggesting that both β_1_- and β_3_-adrenoceptors are involved in the increase in the number of LDs in MCF-7 breast cancer cells.

To further confirm the role of β_1_- and β_3_-adrenoceptors in the LD dynamics in MCF-7 cancer cells, the selective β_1_- and β_3_-adrenoceptor antagonists (atenolol and L-748,337, respectively) were used to investigate whether a blockade of those receptor subtypes would reduce the effects caused by β-adrenoceptor activation on LD area and on LD Nile Red fluorescence intensity. Both atenolol (10 nM) and L-748,337 (100 nM) were able to significantly inhibit the isoprenaline-mediated effects on the area and fluorescence intensity of the LDs (Figure 3B,C, respectively).

### 2.4. Involvement of PKA and EPAC Pathways in the β_1_- and β_3_-Adrenoceptor-Mediated LD Expansion in MCF-7 Breast Cancer Cells

**Figure 4:**
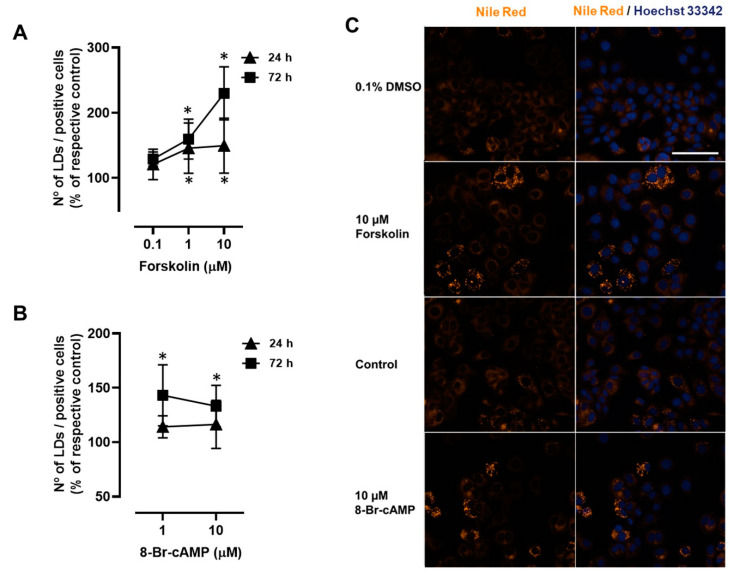
Influence of cAMP-elevating agents forskolin and 8-Br-cAMP on the number of lipid droplets (LDs)/positive cells in MCF-7 breast cancer cells. (**A**) Results for the number of LDs/positive cells in MCF-7 cells after treatment with forskolin (0.1−10 µM) for 24 h or 72 h. (**B**) Results for the number of LDs/positive cells in MCF-7 cells after treatment with 8-Br-cAMP (1 and 10 µM) for 24 h or 72 h. Results are expressed as percentage of control (solvent) and are presented as mean ± SD from 5 independent experiments. * *p* < 0.05, significantly different from solvent. (**C**) Representative microscopic images of LDs (orange fluorescence) in MCF-7 breast cancer cells without any treatment and after treatment, for 72 h, with either 10 µM forskolin or 10 µM 8-Br-cAMP. Nuclei (blue fluorescence) were labelled with Hoechst 33342. Scale bar: 100 µm.

To examine whether cAMP is involved in β-adrenoceptor-mediated LD expansion, MCF-7 cells were incubated with forskolin, which increases intracellular cAMP levels by directly activating adenylyl cyclase. As observed with the adrenaline and isoprenaline, forskolin (0.1–10 µM) also caused a significant time-dependent increase in the number of LDs/positive cells in MCF-7 cells (Figure 4A). An identical experimental approach was performed using the cell-permeable stable cAMP analogue, 8-Br-cAMP. As shown in Figure 4B, 8-Br-cAMP (1–10 µM) also led to a significant increase in the number of LDs/positive cells (only at the 72 h incubation period). Representative images of Nile Red- and Hoechst 33342-stained MCF-7 cells treated with either forskolin or 8-Br-cAMP are shown in Figure 4C.

To further confirm the involvement of cAMP in β-adrenoceptor-mediated LD expansion in breast cancer cells, experiments were carried out in the presence of IBMX (10–30 µM), a non-selective phosphodiesterase (PDE) inhibitor, in order to establish whether the inhibition of PDEs can potentiate β-adrenoceptor-mediated LD expansion in MCF-7 breast cancer cells. Surprisingly, the effects of both adrenaline (100 µM) and isoprenaline (10 µM) were significantly reduced (Figure S3).

**Figure 5:**
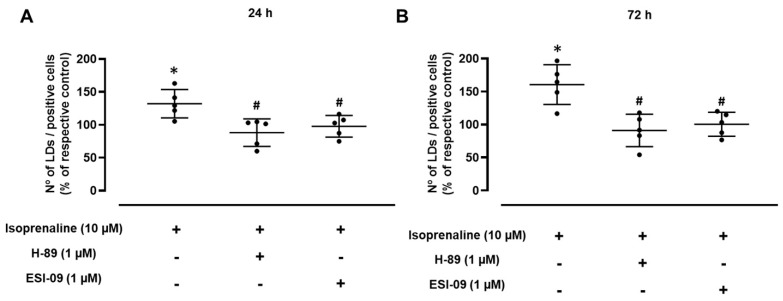
Influence of PKA and EPAC inhibitors, H-89 (1 µM) and ESI-09 (1 µM), respectively, on the effect caused by isoprenaline (10 µM) on the number of lipid droplets (LDs)/positive cells. Cells were treated for (**A**) 24 h or (**B**) 72 h. Results are expressed as percentage of respective controls (cells treated with protein inhibitors alone) and are presented as mean ± SD from 5 independent experiments. * *p* < 0.05, significantly different from solvent; ^#^ *p* < 0.05, significantly different from isoprenaline treatment.

To investigate the involvement of PKA in β-adrenoceptor-mediated LD expansion, cells were treated with isoprenaline (10 µM) in the presence of H-89 (1 µM), a PKA inhibitor. As shown in Figure 5, in the presence of H-89, isoprenaline failed to alter the number of LDs/positive cells after both 24 and 72 h incubation (Figure 5A,B, respectively). The putative involvement of EPAC proteins in the mechanism of LD expansion caused by isoprenaline was also investigated. For this purpose, isoprenaline (10 µM) was tested in the presence of ESI-09 (1 µM), a non-selective inhibitor of EPACs. In the presence of ESI-09, isoprenaline failed to cause any changes in the number of LDs/positive cells after both the 24 and 72 h incubation periods (Figure 5A,B, respectively). Taken together, these results indicate the involvement of both PKA and EPAC proteins in the increase in the number of LDs caused by β-adrenoceptor activation.

## 3. Discussion

LDs are metabolically active organelles that can present periods of expansion, caused by an increase in the accumulation of neutral lipids (mainly TAGs and cholesterol esters) when FAs and cholesterol are abundant in the cytoplasm (LD expansion), and periods of lipid mobilisation of FAs and cholesterol when the cell has higher metabolic needs (LD shrinkage) [20]. These processes can encompass changes in LD size, LD number and/or neutral lipid accumulation, and they are collectively referred to as LD dynamics. Under physiological conditions, LD dynamics are under adrenergic influence through β-adrenoceptor activation [24,25,26,35,45]. In adipocytes, β-adrenoceptor activation induces lipolysis and LD shrinkage [24,25], whereas in pre-adipocytes [46], macrophages [26], hepatocytes [34] and muscle cells [35], β-adrenoceptor activation can induce LD expansion. The present study reveals that, in human MCF-7 breast cancer cells, the activation of β-adrenoceptors alters LD dynamics, promoting LD expansion. To the best of our knowledge, this is the first study to show that β-adrenoceptors can promote LD expansion in cancer cells, revealing a new unexplored mechanism by which β-adrenoceptors may influence the progression of breast cancer.

In cancer cells, LD dynamics may influence cell growth, either by favouring lipid synthesis and storage, when FA amount exceeds cell needs, or lipolysis in order to supply free FAs when metabolic needs are higher. β-adrenoceptor-mediated LD expansion can be caused by an increase in the capacity of cells to uptake lipids, as has previously been shown to occur in brown adipocytes [47], macrophages [26] and the heart [48,49], or by the stimulation of lipogenesis [26]. The experimental approaches used in the present study do not allow for the discrimination of the putative contributions of each mechanism (uptake and lipogenesis), a matter that should be addressed in future studies. Therefore, the β-adrenoceptor-mediated effects on the LD dynamics herein described should be interpreted in a broad sense, that is, as the result of any mechanism that promotes LD expansion.

β-adrenoceptors are a class of G-protein receptors divided into three subtypes: β_1_, β_2_ and β_3_ receptors [50]. All β-adrenoceptor subtypes have been reported to be involved in the mobilisation of LD content [24,51,52] causing LD shrinkage. However, only the β_2_-adrenoceptor subtype has been shown to promote LD expansion [26,45,53]. Surprisingly, in the present study, a contribution to LD expansion by β_2_-adrenoceptors was excluded since salbutamol, a selective β_2_-adrenoceptor agonist, did not cause LD expansion in MCF-7 cells, whereas a clear involvement of both β_1_ and β_3_ subtypes was found, as revealed by the ability of both selective β_1_- and β_3_-adrenoceptor antagonists to block the increase in LDs/cells, LD size and lipid accumulation induced by β-adrenoceptor agonists.

Canonically, β_1_- and β_3_-adrenoceptors are receptors coupled to cAMP signal transduction pathways [50]. cAMP pathways require adenylyl cyclase activation, the subsequent formation of cAMP and the activation of cAMP target proteins [54]. In the present study, the adenylyl cyclase activator forskolin caused LD expansion, supporting the involvement of cAMP in LD expansion. Similar effects of forskolin in LD expansion have been previously reported, namely, in pre-adipocytes [46] and meibocytes [45]. The involvement of cAMP in the β-adrenoceptor-mediated LD expansion was also confirmed by the use of the metabolically stable cAMP analogue, 8-Br-cAMP. The pattern of effects caused by 8-Br-cAMP was not surmountable with those observed by the direct stimulation of adenylyl cyclase, indicating that the cAMP regulation of LD dynamics may be conditioned by the metabolic stability of cAMP.

It is known that cAMP signalling depends on the activity of a family of phosphodiesterases (PDEs) that metabolises cAMP, therefore reducing cAMP-mediated effects or contributing in order to restrict cAMP signalling to specific targets via a compartmentalisation process [55,56]. In the present study, it was shown that phosphodiesterase inhibition did not potentiate but rather prevented the LD expansion caused by the β-adrenoceptor activation. These results support the view that, under the present experimental conditions, PDE activity can compartmentalise cAMP effects towards activating targets involved in LD expansion. The role of PDEs in the compartmentalisation of cAMP signalling has already been demonstrated [55,56,57].

The cAMP targets PKA and EPACs have already been described to influence LD expansion [46,58]. The results of the present study support the involvement of these two cAMP effector proteins in β-adrenoceptor-mediated LD expansion, since this effect was reduced either by H-89, a PKA inhibitor [59], or by ESI-09, a EPAC1/2 inhibitor [60]. Furthermore, the observation that both PKA and EPAC proteins are required to promote LD expansion in human MCF-7 breast cancer cells strongly indicates that the activation of these proteins somehow converge to favour LD expansion. The level at which this convergence occurs remains to be elucidated, but it can be seen as one more example of crosstalk between PKA and EPAC1/2 pathways to provide an integrated control of cAMP signalling [54,61]. The putative sites of convergence may be the transcription factors peroxisome proliferator-activated receptor gamma (PPARγ) and sterol regulatory element-binding protein 1 (SREBP1), previously demonstrated to be downstream targets of cAMP [46,58]. Both PPARγ and SREBP1 are the main regulators of TAG synthesis, and they can activate the expression of genes involved in FA uptake and intracellular transport [62,63,64,65,66]; in the synthesis and esterification of FA [53,67,68,69]; and in the expressions of lipid-droplet-associated proteins, such as perilipins [70].

Although it is known that β-adrenoceptors have relevant effects on breast cancer progression, these effects have been ascribed, to date, mainly to β_2_-adrenoceptors [4,7]. The effects of β_1_- and β_3_-adrenoceptors on breast cancer progression have not yet been studied, even though they are overexpressed in human breast cancer tissues [44]. Based on the present observations, it may be inferred that the LD expansion caused by the activation of β_1_- and β_3_-adrenoceptors may be a mechanism to allow cancer cells to store “energy” and to provide cancer cells with survival advantages, for example, when under nutritional stress [18,39,40], oxidative stress, hypoxia [16] and under antineoplastic therapeutic pressure [41,42]. Indeed, it has been previously reported that LD expansion (revealed by increased levels of the LD-associated protein, perilipin 2) is negatively correlated with relapse-free survival in patients with breast cancer [21]. Therefore, the present results highlight the need for further studies to investigate the importance of β_1_- and β_3_-adrenoceptors in breast cancer and how β_1_-/β_3_-mediated LD expansion influences this disease.

The present study also raises the possibility of adrenergic crosstalk between adipocytes and cancer cells, involving β-adrenoceptor activation. Adipocytes present in the tumour microenvironment have been reported to be a source of FAs for breast cancer cells [71,72]. It is known that β-adrenoceptor activation can promote FA release from adipocytes [24,25]. From the results obtained in the present study, it seems that the adrenergic stimulation that occurs, for instance, under stress conditions, is able to orchestrate the transfer of FAs from adipocytes present in the tumour microenvironment to cancer cells, favouring tumorigenesis. As recently shown by our group [73], cancer cells may acquire the capacity to synthesise noradrenaline and adrenaline and to become a source of autonomous adrenergic stimulation within the tumour microenvironment. Therefore, this orchestrated transfer of FAs from adipocytes may even be generated within the tumour in a sustained way without the need for a general activation of the sympathetic nervous system. This possibility deserves to be further explored, as it could pave the way for the stratification of patients who may better benefit from the use of β-adrenoceptor antagonists to prevent the incidence of breast cancer; that is, this mechanism may be relevant in women with obesity, a population at high risk of breast cancer [74] and with a worse prognosis [74,75].

**Figure 6:**
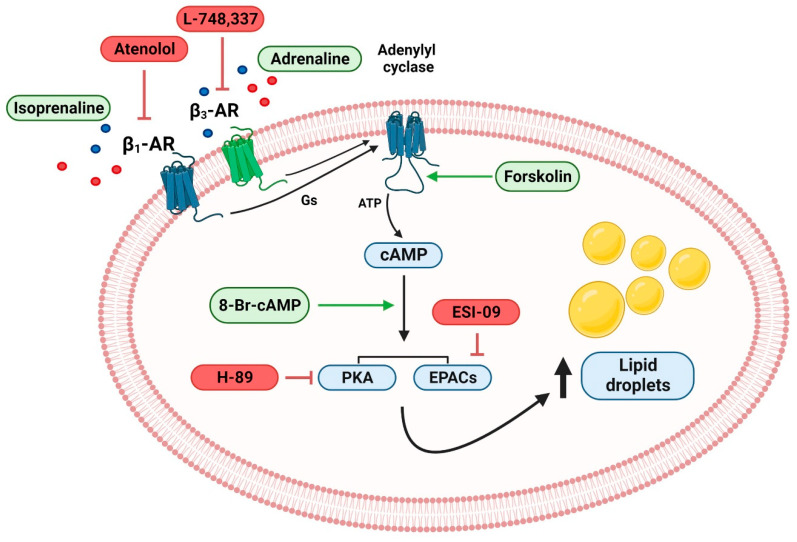
β_1_- and β_3_-adrenoceptors drive lipid droplet expansion in MCF-7 breast cancer cells via cAMP/PKA/EPAC proteins. Drugs tested are shown in green (those used to mimic stimulation of the pathway) and red boxes (those used to block the pathway). AR: adrenoceptor; EPACs: cAMP-dependent guanine-nucleotide-exchange factors; PKA: protein kinase A.

Figure 6 presents the main findings of the current study, which provide a foundation for the understanding of the β-adrenergic contribution to LD dynamics in human breast cancer cells. In conclusion, the present results show, for the first time, that β-adrenoceptors promote LD expansion in human breast cancer cells, a mechanism that may be involved in the adrenergic contribution to breast cancer progression. Giving the relevance of clinically exploring the claimed effects of β-blockers in reducing breast cancer progression, further studies are needed to confirm the relevance of the β-adrenoceptor-mediated LD expansion in human breast cancer cells and to determine whether blocking this pathway may be clinically relevant in preventing breast cancer in the obese population.

## 4. Materials and Methods

### 4.1. Chemicals

(−)-Adrenaline bitartrate, (−)-isoprenaline hydrochloride, salbutamol, atenolol, L-748,337, ESI-09, forskolin, 3-Isobutyl-1-methylxanthine (IBMX), 8-Bromoadenosine 3′,5′-cyclic monophosphate (8-Br-cAMP), H-89 dihydrochloride hydrate, Nile Red and penicillin/streptomycin were obtained from Sigma-Aldrich (Chemicalnor, Valongo, Portugal). Foetal bovine serum (FBS) was obtained from Biochrom (Biotecnómica, São Mamede, Portugal). Dulbecco’s modified Eagle’s medium—high glucose, supplemented L-Glutamine (Gibco) and 0.25% trypsin/0.025% EDTA solution (Gibco) were obtained from Alfagene (Carcavelos, Portugal).

### 4.2. Cells, Culture Conditions and Treatments

MCF-7 cells (HTB-22; ATCC, LGC standards, Barcelona, Spain) were cultured in Dulbecco’s modified Eagle’s medium (DMEM) with 4.5 g/L of glucose, 10% heat-inactivated FBS, 100 μg/mL of streptomycin and 100 U/mL of penicillin. The cells were cultivated at 37 °C in a humidified atmosphere of 95% air and 5% CO_2_ and below 90% confluence. Subculturing was performed twice a week via trypsinisation (0.25% trypsin/0.025% EDTA).

In each experimental assay, the trypsinised MCF-7 cells were centrifuged at 400× *g* for five minutes at 20 °C. Viable cells were counted using the trypan blue exclusion method and subsequently seeded at a cell density of 2.3 × 10^4^ cells/mL. Depending on the experimental goal, the cells were treated with either isoprenaline (0.1–10 µM), adrenaline (1–100 µM), salbutamol (0.1–10 µM), atenolol (10 nM), L-748,337 (100 nM), forskolin (0.1–10 µM), 8-Br-cAMP (1,10 µM), H-89 (1 µM), ESI-09 (1 µM) or IBMX (10, 30 µM) alone or in combination and incubated for up to 72 h. DMSO (maximum concentration used of 0.1% *v/v*) was also included in each experimental assay.

### 4.3. Nile Red Staining

LD labelling was performed at the end of the different incubation periods (24 h and 72 h) using Nile Red staining, according to the protocol described by Dates, Fahmi [76]. Briefly, the MCF-7 cells were fixed with 4% paraformaldehyde for 10 min, washed with PBS and incubated with Nile Red (10 µg/mL in PBS) for 15 min at room temperature. The cells were then washed twice with PBS and incubated with a solution of Hoechst 33342 (5 μg/mL) for 30 min for nuclei staining. The cells located in the centre of the well were imaged using a Lionheart FX automated microscope (Biotek Instruments, Winooski, VT, USA) with a 20× objective lens. RFP and DAPI channels were used to capture images of both the stained neutral lipids (included in LDs) and nuclei, respectively. The stitching of images was performed using the linear blend fusion method, whereas background removal was performed using the rolling ball algorithm in Gen5 3.04 software. For image quantification purposes, the freely available Cellprofiler^TM^ image analysis software was used. Cell nuclei segmentation was performed in the stitched DAPI-acquired images using the minimum cross-entropy thresholding method [77], whereas cell boundary segmentation was performed in the stitched RFP-acquired images using the Otsu thresholding method [78]. For LD segmentation, post-processed (stitched and removed background) RFP images were used and analysed using a manual threshold method. Proper segmentations were manually verified for each condition tested using the Overlay outlines module. The calculations of the area and Nile Red fluorescence intensity per LD and the percentage of cells presenting LDs (% of positive cells) were carried out using the respective Cellprofiler^TM^ modules.

In independent experiments, the % of positive cells varied between 50 and 90% but, within the same experiment, the fraction of positive cells in the non-treated MCF-7 cells was similar. The average number of LDs/positive cells was similar at 24 h and 72 h incubation, indicating that LD dynamics are not affected by the length of the incubation time. This further indicates that, in the present experimental conditions, the number of LDs/positive cells is not influenced by cell density (higher at 72 h).

### 4.4. Statistical Analysis

The results are presented as mean ± SD. GraphPad Prism 8 software was used to design graphs and to perform statistical analyses. Differences between treatments and controls were compared using two-way ANOVA with repeated measures, followed by the post hoc multi-comparisons Dunnett’s *t* test, unless otherwise stated. Differences between treatments or between distinct incubation periods were evaluated using Student’s *t*-test.

## Data Availability

The data presented in this study are available on request from the corresponding author.

## Supplementary Materials

The following supporting information can be downloaded at: https://www.mdpi.com/article/10.3390/ijms24010767/s1.
