# Supplementary material for: Activation of β-Adrenoceptors Promotes Lipid Droplet Accumulation in MCF-7 Breast Cancer Cells via cAMP/PKA/EPAC Pathways
Source: Int J Mol Sci. 2023 Jan 1;24(1):767. doi: 10.3390/ijms24010767 (PMC9820888; doi:10.3390/ijms24010767)
Supplement: Supplementary file 1 [file ijms-24-00767-s001.zip › ijms-2116903-supplementary.pdf]

Supplementary Materials

Activation of β-adrenoceptors promotes lipid droplet accumulation in MCF-7 breast cancer cells via cAMP/PKA/EPAC pathways

Dany Silva[1,2], Katarzyna Kacprzak[1], Clara Quintas[1,2], Jorge Gonçalves[1,2*], Paula Fresco[1,2]

[1] Laboratory of Pharmacology, Department of Drug Sciences, Faculty of Pharmacy, University of Porto, Porto, Portugal

[2] UCIBIO-REQUIMTE, Department of Drug Sciences, Faculty of Pharmacy, University of Porto, Porto, Portugal

***** Correspondence: jgoncalves@ff.up.pt

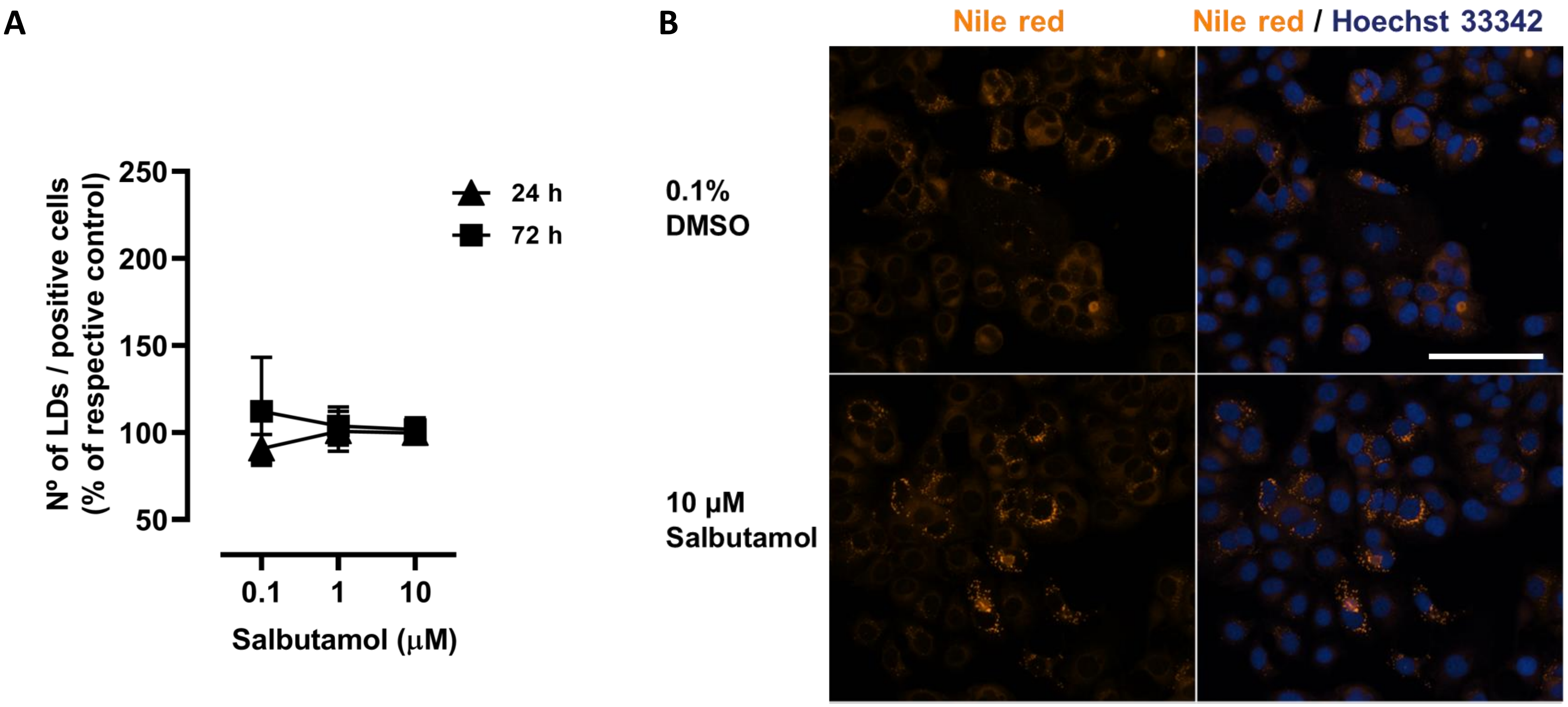

Figure S1. Influence of β_2-adrenoceptor activation on the number of lipid droplets (LDs)/positive cells in MCF-7 breast cancer cells. Cells were treated with the β_2-adrenoceptor agonist salbutamol (0.1-10 μM) (A) for 24 h or 72 h. Results are expressed as percentage of control (solvent) and are presented as mean ± SD from 4-5 independent experiments. (B) Representative microscopic images of LDs (orange fluorescence) in MCF-7 breast cancer cells after treatment with 10 μM salbutamol for 72 h. Nuclei (blue fluorescence) was labelled with Hoechst 33342. Scale bar: 100 μm.

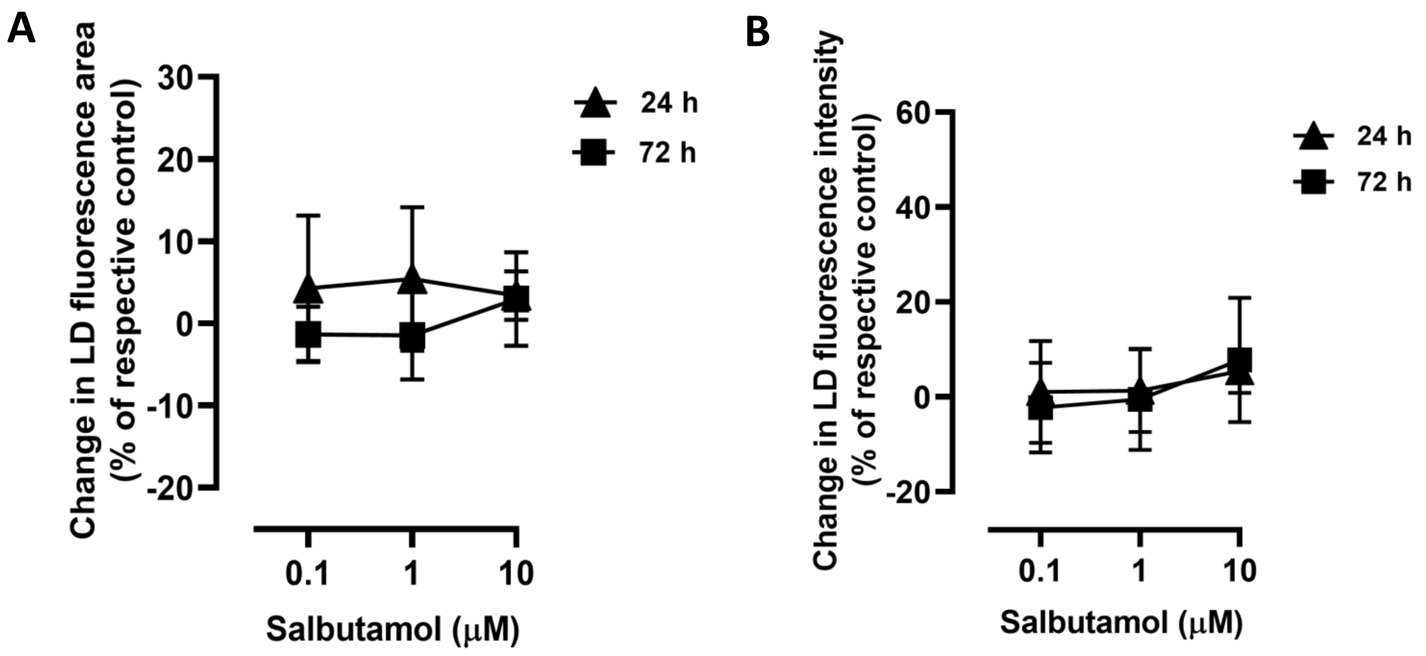

Figure S2. Influence of β_2-adrenoceptor activation in the (A) area and (B) Nile Red fluorescence intensity of lipid droplets (LDs) in MCF-7 breast cancer cells. Cells were treated with selective β_2-adrenoceptor agonist salbutamol (0.1-10 μM) for 24 h or 72 h. Results shown are a percentage of change in LD area or in LD fluorescence intensity comparatively to control (solvent) and are presented as mean ± SD from 4-5 independent experiments.

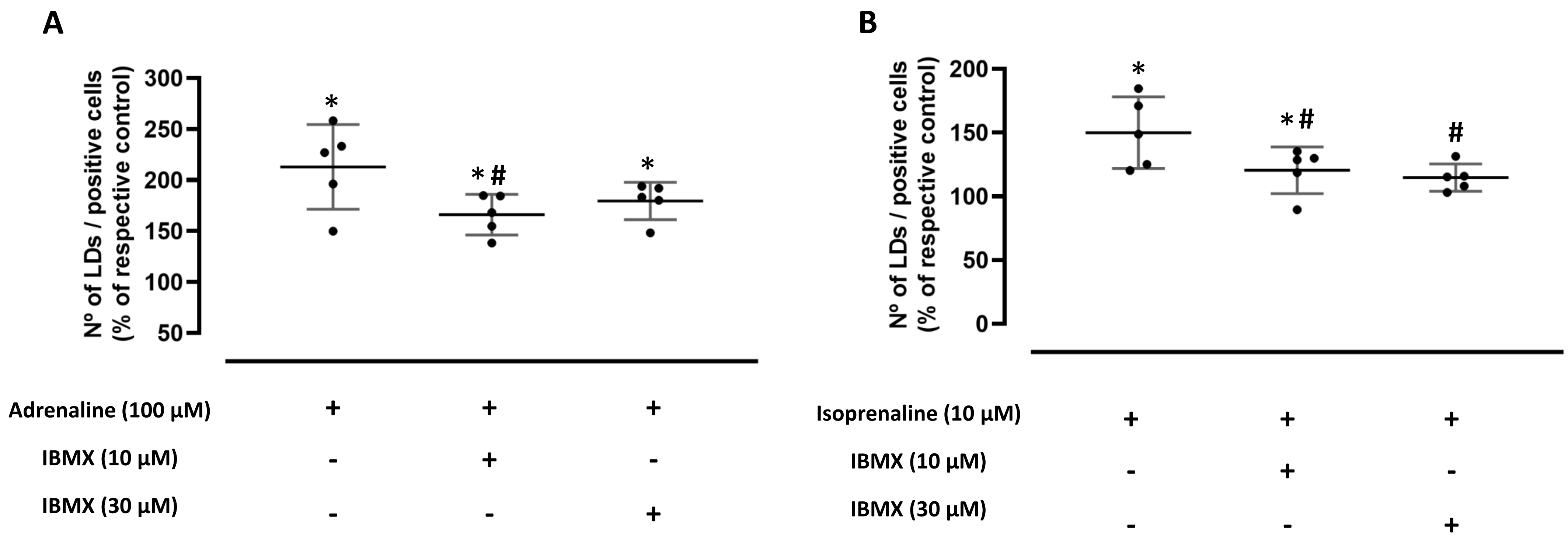

Figure S3. Influence of the non-selective phosphodiesterase inhibitor, IBMX (10 and 30 µM) in the effects caused by (A) adrenaline (100 µM) or by (B) isoprenaline (10 µM) on the number of lipid droplets (LDs)/positive cells after 72 h of incubation. Results are expressed as percentage of control (solvent) and are presented as mean ± SD from 5 independent experiments. *$p<0.05$, significantly different from solvent; $^{\#}p<0.05$, significantly different from isoprenaline treatment.
